# Effectiveness of a multicomponent exercise intervention in community-dwelling older Chinese people with cognitive frailty: protocol for a mixed-methods research

**DOI:** 10.3389/fnagi.2024.1282263

**Published:** 2024-02-12

**Authors:** Hongting Ning, Fenghui Chen, Junxin Li, Yan Du, Xi Chen, Shuang Wu, Abigael Joseph, Yinyan Gao, Zeng Cao, Hui Feng

**Affiliations:** ^1^Xiangya School of Nursing, Central South University, Changsha, Hunan, China; ^2^School of Nursing, Johns Hopkins University, Baltimore, MD, United States; ^3^Nursing School, Xinjiang Medical University, Urumqi, China; ^4^School of Nursing, The University of Texas Health Science Center at San Antonio, San Antonio, TX, United States; ^5^Department of Epidemiology and Health Statistics, Xiangya School of Public Health, Central South University, Changsha, Hunan, China; ^6^Department of Physical Medicine and Rehabilitation, Xiangya Hospital, Central South University, Changsha, Hunan, China

**Keywords:** exercise intervention, exergaming, resistance exercise, community-dwelling, older adults, cognitive frailty, mixed methods

## Abstract

**Aims:**

To evaluate the effectiveness of a multicomponent exercise intervention and to clarify the underlying mechanisms of the program in community-dwelling older adults with cognitive frailty. Additionally, the perception of participants in the program will be explored.

**Design:**

A mixed-methods design, including a randomized controlled trial and an exploratory qualitative study, was used.

**Methods:**

Each group consists of 41 participants. The experimental group will undergo a 12-week multicomponent exercise intervention, including warm-up, exergaming aerobic exercise, elastic-band resistance exercise, and cool-down. This intervention was developed based on the Health Belief Model (HBM) and Self-Efficacy Model (SEM). The control group will not receive any intervention. Physical frailty and cognitive function will be considered as primary outcomes. Data will be collected both at baseline and at the end of the intervention period. Fisher’s exact test, analysis of covariance, and generalized linear models will be conducted to compare mean changes between the two groups. Additionally, the mediation models will be used to examine whether any intervention effects are mediated through exercise self-efficacy.

**Discussion:**

The findings of this study are anticipated to provide valuable insights for healthcare providers, enabling them to learn about effective strategies to enhance exercise adherence and promote improved functionality, independence, and quality of life for older adults with cognitive frailty.

Clinical trial registration: [https://clinicaltrials.gov/], identifier [ChiCTR2200058850].

## 1 Introduction

Physical frailty emerges as a prevalent geriatric syndrome globally, which is characterized by age-associated declines in physiologic reserve and function across multiorgan systems (Fried et al., 2001). One of the most common physical frailty models described in the literature is the Fried frailty phenotype, which defines frailty as a clinical syndrome meeting three or more of the five indicators: unintentional weight loss, physical inactivity, exhaustion, weakness, and slowness (Fried et al., 2001). One recent systematic review provides prevalence proportions for older adults in population-level studies from 62 countries/territories for using the Fried frailty phenotype approaches to define frailty, generating a pooled prevalence of 12% for physical frailty (O’Caoimh et al., 2020). Physical frailty overlaps with other geriatric syndromes, such as cognitive impairment, resulting in increased vulnerability to various adverse health outcomes (Kelaiditi et al., 2013; Chu et al., 2021).

Cognitive frailty has been defined as the presence of both physical frailty and cognitive impairment without a clinical diagnosis of Alzheimer’s disease and other dementias (Kelaiditi et al., 2013). The prevalence of cognitive frailty among community-dwelling older adults is 6% (Zhang et al., 2022). Compared to either physical frailty alone or cognitive impairment alone, cognitive frailty poses a higher vulnerability to adverse outcomes, including disability, dementia, and mortality (Kelaiditi et al., 2013; Ruan et al., 2015). Cognitive frailty has been considered an intervention window because older adults with cognitive frailty are more likely to revert to a robust state through appropriate interventions compared to those with dementia and disability (Solfrizzi et al., 2017). Research has shown that exercise intervention is a highly recommended strategy for improving the health outcomes of older adults with cognitive frailty (Chen et al., 2021). Exercise, as a subset of physical activity, is characterized by planned, structured, and repetitive implementation (Caspersen et al., 1985; Thivel et al., 2018). It aims to improve or maintain one or more components of physical fitness (Dasso, 2019). Exercise can be performed in different forms and can differ in intensity, duration and type (e.g., aerobic and strength). According to the American College of Sports Medicine (ACSM) recommendations, multicomponent physical activity, including both aerobic and muscle-strengthening activities, should be adopted among older adults (Nelson et al., 2007).

## 2 Background

Older adults with cognitive frailty are acknowledged to be frailer compared to their healthier peers. Furthermore, they tend to have sedentary lifestyle and engage less in physical activity (Kwan et al., 2020). Research has indicated that a sedentary lifestyle and physical inactivity are associated with adverse health outcomes (Theou et al., 2017). Conversely, physical exercise may enhance physical performance, cognitive function, and mental health in individuals with cognitive frailty (Yoon et al., 2018; Chen et al., 2021; Li et al., 2022). This highlights the importance of promoting physical exercise within this group.

However, it is challenging to change people’s physical exercise behaviors. Adherence to physical exercise interventions is often low among older population (Valenzuela et al., 2018; Moral-Munoz et al., 2022). Exercise adherence plays a crucial role in determining whether and how much the exercise intervention could be effective. Some exercise interventions have failed to achieve the intended effectiveness due to poor exercise adherence (Clegg et al., 2014; Gwyther et al., 2018). Previous studies have identified common reasons for this lack of adherence, such as physical limitations, environmental barriers, and time constraints (André and Agbangla, 2020; Collado-Mateo et al., 2021). Additionally, lack of motivation and enjoyment of exercise have been recognized as significant factors influencing adherence (André and Agbangla, 2020; Collado-Mateo et al., 2021).

Exergaming, a form of enjoyable aerobic exercise-based video games that combine physical activity and cognitive stimulation within a virtual environment, has emerged as an innovative way to encourage exercise among older adults (Choi et al., 2017; Karssemeijer et al., 2019). Several studies have examined the adherence and effectiveness of exergaming in older adults, revealing promising results that underscore its ability to enhance motivation for sustaining exercise programs (Lamoth et al., 2011; Altamimi and Skinner, 2012). However, while these programs target older people in general, they do not focus on older adults with cognitive frailty (Cacciata et al., 2019; Yen and Chiu, 2021). Additionally, these exergaming programs typically focus solely on aerobic activities, lacking of muscle-strengthening exercises, which may potentially limit their overall health benefits (Stojan and Voelcker-Rehage, 2019; Esmaeilzadeh et al., 2022). The ideal exercise prescription for older individuals should incorporate both aerobic and muscle-strengthening exercise (Venegas-Sanabria et al., 2022). Both forms of exercise are crucial for successful performance in activities of daily living (ADLs) and for maintaining physical and psychological wellbeing (Chodzko-Zajko et al., 2009). Relying solely on a single form of exercise may not provide comprehensive benefits (Chodzko-Zajko et al., 2009).

Furthermore, the selection of assessments is pivotal in intervention studies. Previous studies among cognitive frailty have predominantly focused on intervention effectiveness related to frailty, cognitive function, muscle-related outcomes, physical functional abilities, and quality of life. However, there’s been a lack of attention to intervention effects on senior fitness, sleep quality, and mental health (Yoon et al., 2018; Kwan et al., 2020). Researchers are encouraged to explore the effectiveness of exercise interventions from a broad perspective for comprehensive effectiveness evaluation, considering exercise interventions have been proven to offer a wide range of health benefits (Qiao et al., 2022).

**Figure 1:**
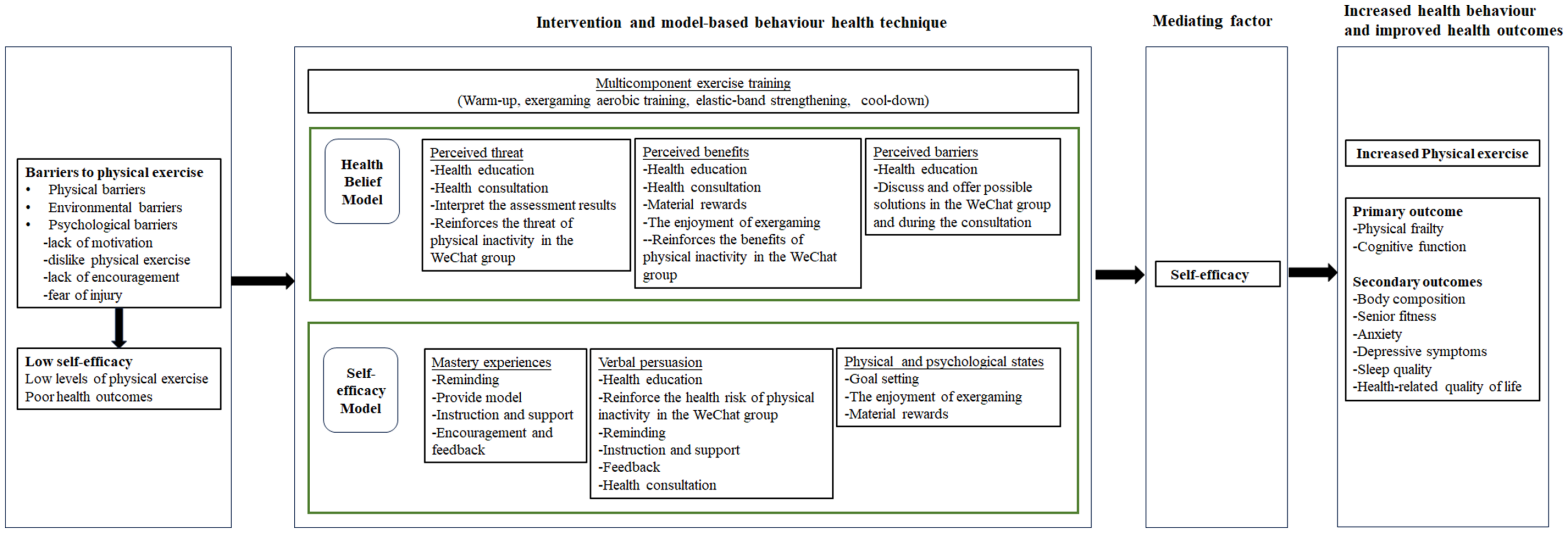
Conceptual framework for the intervention.

Moreover, most previous exercise studies lack a theoretical basis for understanding exercise behavior change (Hauer et al., 2020; Huang, 2020), thereby limiting the exploration of potential intervention mechanisms among older adults with cognitive frailty. Currently, the Health Belief Model (HBM) and the Self-Efficacy Theory (SET) are two commonly used theoretical frameworks in physical exercise research (Biddle and Nigg, 2000). Our proposed intervention program is guided by theoretical constructs drawn from the HBM and SET (Figure 1). Self-efficacy, defined as one’s confidence in performing particular behaviors, significantly influences health behavior performance, subsequently impacting health outcomes (Bandura, 1994). Perceived threat, perceived benefits, perceived barriers, mastery experience, verbal persuasion, and physical and psychological states stand as key sources contributing to self-efficacy (Bandura, 1994). Studies have shown that self-efficacy predicts physical exercise behavior in older adults and plays a role in both the adoption and maintenance of physical exercise (Di Maio et al., 2021; Bateman et al., 2022). Additionally, experimental findings support the idea that changes in self-efficacy can act as a mediator in the impact of behavior interventions on objectively measured physical exercise behavior (Ratz et al., 2020).

The proposed intervention aims to enhance the participant’s self-efficacy by employing a series of model-based behavior health technique. Techniques such as reminders, goal setting, encouragement, and feedback, when coupled with incentives, serve as motivators to improve self-efficacy (Ashford et al., 2010; Eather et al., 2013; Lewis et al., 2016). Consequently, the intervention program, incorporating multicomponent exercise training and model-based behavior health technique, is hypothesized to provide older adults with a stronger sense of self-efficacy, leading to increased physical exercise and improved health outcomes.

Therefore, the objectives of this study were: (1) to evaluate the effectiveness of a multicomponent exercise intervention in improving the health outcomes (physical frailty, cognitive function, body composition, senior fitness, mental health, sleep quality, and health-related quality of life) among older adults with cognitive frailty; (2) to examine whether any health-improving effects are mediated through exercise self-efficacy; and (3) to explore the participants’ perception of the multicomponent exercise intervention.

## 3 Methods

### 3.1 Research hypotheses

To assess the impact of the multicomponent exercise intervention, our study hypothesizes several outcomes across physical, cognitive, mental, and quality-of-life domains. We aim to explore the potential effects of this intervention on various parameters and examine the mediating role of exercise self-efficacy.

(1) We hypothesized that participants who receive the multicomponent exercise intervention will report greater improvements in physical frailty, cognitive function, body composition, senior fitness, mental health, sleep quality, and health-related quality of life upon completion of the intervention compared to those in the control group.
(2) We hypothesized that the effects of the multicomponent exercise intervention on physical frailty, cognitive function, body composition, senior fitness, mental health, sleep quality, and health-related quality of life are mediated through an increase in exercise self-efficacy.
(3) No starting hypothesis has been established for the qualitative part of the study because it is oriented through an exploratory study.

### 3.2 Methodology

#### 3.2.1 Study design, setting, and participants

This mixed methods study comprises a single-blind (outcomes assessor) randomized controlled trial (RCT) and a descriptive qualitative study. It will be conducted in community settings targeting older adults with cognitive frailty in Changsha City, Hunan Provence, China. Community settings will be chosen based on approval from local committees and the availability of eligible older adults. To engage older residents and encourage their participation, we will utilize posters, flyers, and social media platforms to disseminate information about the study. Additionally, recruitment will be conducted in collaboration with healthcare personnel who are responsible for the preventive home visits for the local residents.

Inclusion criteria:

(1) Aged ≥60 years.
(2) Fulfilling the definition of cognitive frailty, which includes:
(a) pre-physical frailty or physical frailty (Fried frailty phenotype score ≥ 1), and.
(b) Montreal Cognitive Assessment (MoCA) scored between 18 and 25, but in the absence of dementia (Kelaiditi et al., 2013).
(3) Providing informed consent and voluntary participation in the study.

Exclusion criteria:

(1) Poorly controlled or severe status of cardiovascular diseases, chronic obstructive pulmonary disease, diabetes, or hypertension.
(2) Inability to walk independently.
(3) Severe hearing or visual impairments.
(4) Other health conditions that prevent participation in sports exercise.
(5) Currently enrollment in other intervention programs.

**Figure 2:**
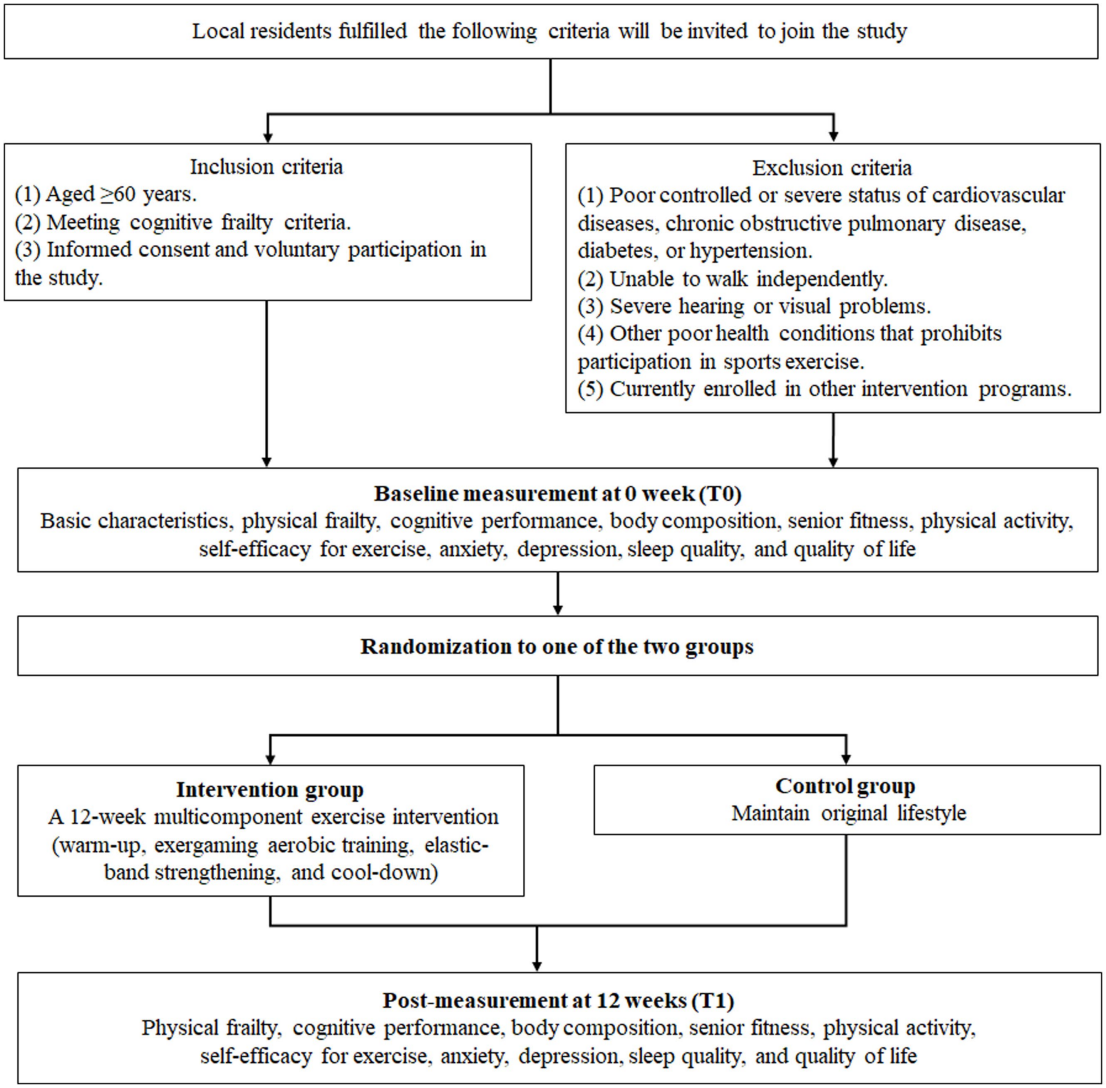
Study design.

The trial will run for a 12-week period. Participants will be enrolled based on inclusion and exclusion criteria. Measurements will be conducted at baseline and at the end of the intervention period. An overview of the study design is presented in Figure 2. Regarding the qualitative study, a subgroup of participants assigned to receive the multicomponent exercise intervention will undergo semi-structured interviews to explore their perception of the program.

#### 3.2.2 Intervention

Based on literature review, interviews with cognitive frail older adults, and consultations with physiotherapist, a preliminary multicomponent exercise intervention has been recommended for older adults with cognitive frailty. During the 12-week multicomponent exercise intervention period, participants will engage in moderate physical exercise tailored to their individual abilities, with each session lasting 50 min, held 3 times per week in a group setting at the local community center. Trained research assistants will provide assistance during these sessions. To control for concomitant exercise and diet, all participants will be advised not to seek any other exercise and instructed to maintain their normal diets throughout their enrollment in the study.

The daily session will be divided into warm-up, exergaming aerobic exercise, elastic-band resistance exercise, and cool-down. Each session will start and end with a 5-min warm-up and cool-down routine to stretch the main muscle groups being trained. Ping-Pong exergaming on the Xbox 360 4GB Console with Kinect will be chosen as the aerobic exercise because Ping-Pong is familiar to most older adults in China and requires fewer skills compared to other types of exergaming. This type of exergaming is suitable for older adults with cognitive frailty because it is easier to learn. The progression of ping-pong exergaming will be managed by adjusting the exercise intensity. The duration of the ping-pong exergaming would be approximately 20 min per daily session. The intensity will be gradually increased throughout the entire intervention period based on the self-reported tiredness and assessment conducted by trained exercise supervisors. Resistance exercise will utilize an elastic band (Thera-Band elastic band). The general recommendation will be an exercise intensity of 70–80% of 1 repetition maximum (1RM) allowing older adults to perform 8–10 repetitions (Liguori and American College of Sports Medicine, 2020). The duration of the resistance exercise will be about 20 min per session. The elastic-band is color-coded, with different colors representing different levels of resistance. In this study, yellow, red, green, and blue elastic-bands will be used alternately to increase resistance intensity. To ensure participants exercise at the appropriate intensity, their perceived effort levels will occasionally be assessed using the Borg Rating of Perceived Exertion scale, which ranges from 6 to 20 (Borg, 1982). Participants will be asked to report their average intensity level during the exercise sessions. Depending on the participant’s fitness level, exercises should be performed with a perceived exertion range between 9 and 16 points during both aerobic and resistance training (Borg, 1982). Further details are presented in Table 1. The final exercise intervention protocol will be determined based on a pilot study involving 10 participants to assess feasibility.

**Table 1:** Description of the multicomponent exercise intervention.

Category	Description
Warm-up	Freehand training range of motion exercise for the wrists, hip, shoulder, knees, and ankles (about 5 min)
Aerobic exercise	Exergaming-based Ping-Pong aerobic exercise involves dynamic body movements, including swinging motions with virtual Ping-Pong paddles, upper body rotation to hit the ball accurately, and leg movements to position oneself correctly (about 20 min)
Resistance exercise	The action includes elastic-band pull-apart, front and lateral raises, elbow flexion/bicep curl, and elastic-band lateral leg abductions, etc. (about 20 min)
Cool-down	Freehand stretching and relaxing exercises involve the main muscles and joints of the whole body (about 5 min)

In the control group, participants will maintain their normal daily activities without receiving any special intervention. Throughout the 12-week intervention period, if participants engage in additional physical activity, they will be required to document the details, including the activity time, type, and intensity. To avoid cross-contamination between the two groups, control over the exergaming power switch will remain with the intervention team, not the participants. Additionally, the elastic bands provided during sessions will be collected afterwards rather than kept by the participants.

#### 3.2.3 Strategies to support behavior change

To enhance adherence to the multicomponent exercise intervention, strategies supporting behavior change will be implemented, integrating elements from the HBM and SET. Previous studies suggest that exercise interventions among older adults should prioritize strengthening their beliefs and self-efficacy. This involves educating them about the negative consequences of physical inactivity, highlighting the benefits of exercise, and teaching coping strategies to overcome potential barriers. It also focuses on helping participants mastery exercise experiences, maintain good physical and psychological states, and employ persuasion strategies (Biddle and Nigg, 2000; McAuley and Blissmer, 2000; Glanz et al., 2015). Details of the behavior change techniques (BCTs) (Michie et al., 2011) and how they are incorporated into this exercise intervention program are presented in Table 2.

**Table 2:** Behavior change techniques used in this exercise intervention program.

Beliefs and self-efficacy	Behavior change technique	How the technique is in this intervention
Perceived threat (HBM)	Provide information on consequences of behavior in general	Health education, interveners give information about correlation between insufficient physical activity and potential adverse effects in the general case. Interveners reinforce the health risk of physical inactivity through WeChat group.
	Provide information on consequences of behavior to the individual	Interveners interpret the baseline assessment results for participants, describe to the participants the underlying impairments identified at the baseline assessment. Interveners reinforce the health risk of physical inactivity during the consultation.
Perceived benefits (HBM)	Provide information on consequences of behavior in general	Health education, interveners disseminate knowledge regarding the health benefits of physical activity. Interveners reinforce the health benefits of physical activity through WeChat group.
	Provide information on consequences of behavior to the individual	Interveners interpret the baseline assessment results for participants, describe to the participants the health benefits of physical activity.
Perceived barriers (HBM)	Barrier identification /problem solving	Interveners ask the participants possible barriers and problems, discuss and offer possible solutions in the Wechat group and during the consultation. A brief medical safety check before each session to reduce injury risk.
Mastery experiences (SET)	Provide instruction on how to perform the behavior	Provide instruction and support on how to use exergaming equipment and elastic-band. Tips on wearing suitable clothing, shoes, and avoiding empty stomach, reminding when and where of each session will be sent to WeChat group.
	Model/Demonstrate the behavior	Each session will have a model showing the participants how to perform the multicomponent exercise training.
	Facilitate social comparison	Interveners will encourage participants to communicate and compare with other participants during training.
	Action planning	At week 1, interveners tell participants of detailed planning of what they will do including when, where, and how to act.
Physical and psychological states (SET)	Goal setting	At week 1, interveners and participants set individual goals concerning exercise training based on consider of participants’ physical and psychological states.
	Stimulate anticipation of future rewards	Participants are informed at the beginning (week 1) that they will be rewarded based on their behavioral achievement.
Verbal persuasion (SET)	Provide information on consequences of behavior in general	Health education, interveners give information about correlation between insufficient physical activity and potential adverse effects in the general case. Interveners reinforce the health risk of physical inactivity through WeChat group.
	Provide feedback on performance	Participants will receive real-time feedback when playing exergaming. Intervener praise participants if they behave well during the training.
	Provide instruction on how to perform the behavior	Provide instruction and support on how to use exergaming equipment and elastic-band. Tips on wearing suitable clothing, shoes, and avoiding empty stomach, reminding when and where of each session will be sent to WeChat group.
	Provide normative information about others’ behavior	Intervener provide information about other’s good performance and encourage individuals to learn from successful experience through WeChat group.
	Prompt review of outcome goals	Intervener helps participants to review or analysis of the extent to which previously set outcome goals were achieved during the consultation.

#### 3.2.4 Sample size estimation

For the RCT, the sample size will be calculated using PASS software, based on improvement in the primary outcome (physical frailty) in this trial. Previous findings from a similar exercise program reported a reduction in physical frailty score of 0.22 in the control group and 1.59 in the intervention group (Sadjapong et al., 2020). Given a power of 90% and level *α* = 0.05, 34 participants are needed. Considering a 20% attrition rate, with 41 participants in each group, a total of 82 participants are needed. Regarding the qualitative interviews, a purposive subsample of 20 participants who have completed the multicomponent exercise intervention will be recruited.

#### 3.2.5 Randomization and blinding

A biostatistician, not involved in the trial, will randomize all eligible participants using a computer-generated random number list. Group assignments will be concealed in consecutively numbered sealed envelopes that will be opened sequentially upon the enrollment of each participant. Eligible subjects will be randomly allocated into two groups: the multicomponent exercise group and the control group. Participants and interveners will not be blinded to the intervention assignment due to the nature of exercise intervention. However, outcome assessors and data analysts will be blinded to group assignments.

#### 3.2.6 Measurement

##### 3.2.6.1 RCT

The measurements in this trial will consist of basic characteristics, physical frailty, cognitive performance, body composition, senior fitness, self-efficacy for exercise, anxiety, depressive symptoms, sleep quality, and health-related quality of life. Exercise self-efficacy will be chosen as the mediating factor. Physical frailty and cognitive function will be considered as the primary outcomes, while the rest are secondary outcomes. All these variables will be assessed at baseline and at the end of the intervention period. Additionally, we will collect data on adherence, dropout rate, satisfaction, and adverse events as complementary measures. Trained research assistants specializing in geriatrics will conduct these assessments.

###### 3.2.6.1.1 Basic characteristics

Participants’ demographic characteristics (e.g., age, sex, education, marital status, living arrangements, occupation, and income), lifestyle information (drinking and smoking), comorbidities, and medication use will be collected using a self-designed questionnaire.

###### 3.2.6.1.2 Mediating factor

The Self-Efficacy for Exercise (SEE) Scale is a self-report of exercise self-efficacy. This scale has a range of total scores from 0–90. A higher score indicates higher self-efficacy for exercise (Resnick and Jenkins, 2000).

###### 3.2.6.1.3 Primary outcomes

Physical frailty will be measured according to the Cardiovascular Health Study criteria (Fried et al., 2001) to enable comparability. The assessment includes five components:

- Shrinking: Determined by unintentional weight loss exceeding 4.5 kg or more than 5% of body mass in the past 12 months.
- Weakness: Assessed through Body mass index (BMI) and sex-adjusted handgrip strength. Men cut-off points: 29, 30, and 32-kg handgrip strength for normal-weight, overweight and obese, respectively. Women: 17, 17.3,18, and 21 kg for underweight, normal-weight, overweight and obese, respectively.
- Exhaustion: Assessed by the frequency of fatigue in the last week using two items of the Center of Epidemiological Studies-Depression Scale (CES-D) (Orme et al., 1986).
- Slowness: Determined by height- and sex-adjusted gait speed. Cut-off points are 0.65 m/s of walking speed over a 4.57-m distance for men and women ≤173 cm and ≤ 159 cm, respectively, and 0.76 m/s for height above these ranges.
- Low physical activity: Assessed using the International Physical Activity Questionnaire-Short Form (IPAQ-SF). Energy expenditure (kcal/week) will be calculated for each participant, and low physical activity will be identified by an expenditure of <383 kcal/week in men and < 270 kcal/week in women (Brunner et al., 2018; Piotrowicz et al., 2023).

Individuals will be categorized into three groups based on their total score: robust (0), prefrail (1–2), and frail (3–5) groups (Fried et al., 2001). For this study, frailty status will be classified into two groups: the frail group (1–5) and the robust group (0).

The Montreal Cognitive Assessment (MoCA) is a brief screening tool for global cognition that reveals mild cognitive impairment and an early stage of Alzheimer’s disease (Nasreddine et al., 2005). This tool evaluates various cognitive domains, including attention, concentration, executive function/visuospatial ability, memory, language, conceptual thinking, calculations, and orientation. MoCA scores range from 0 to 30, with scores of 26 and higher generally considered normal. Scores falling between 25 and 18 indicate mild cognitive impairment, while scores lower than 18 are indicative of Alzheimer’s disease (Nasreddine et al., 2005). The Chinese version of MoCA (Beijing version) is widely utilized in China due to its established validity, reliability, and sensitivity (Yu et al., 2012).

###### 3.2.6.1.4 Secondary outcomes

Body composition assessment will be conducted using the InBody S10. BMI will be calculated by dividing weight in kilograms by the square of height in meters. The senior fitness test battery referred to *Senior Fitness Test Manual, Second Edition* (Rikli and Jones, 2013), will be performed in the following order to minimize fatigue: (1) chair stand test (lower-body muscular strength); (2) arm curl test (upper-body muscular strength); (3) chair sit-and-reach test (flexibility); (4) back scratch test (flexibility); (5) 2.4-meter up-and-go test (dynamic balance); (6) single leg stand test (dynamic balance); and (7) 2-min step test (aerobic endurance). Anxiety will be assessed using the Generalized Anxiety Disorder Scale (GAD-7) (Dear et al., 2011), while depressive symptoms will be assessed using the Patient Health Questionnaire-9 (PHQ-9) (Löwe et al., 2004). Sleep quality will be determined by the Pittsburgh Sleep Quality Index (PSQI) (Buysse et al., 1989), and health-related quality of life will be measured using the validated Chinese version of the Short Form Health Survey questionnaire (SF-12) (Ware et al., 1996). All these assessment tools have been demonstrated to have relatively good reliability and validity when used with the Chinese population.

###### 3.2.6.1.5 Other collected data

Adherence to the program will be documented via an activity diary completed by the research assistants, and reasons for dropouts and poor adherence will also be documented. Reasons for dropout, including adverse events, medical problems, death, and participant decisions, will be collected. Program satisfaction will be evaluated using a self-designed questionnaire. Participants will rate their satisfaction based on perceived enjoyment of the training, the appropriateness of the content, the form of the training, the benefits obtained by the training, and the likelihood of recommending the program to others. Rating will use a 4-point Likert scale, with “1” indicating “not at all” and “4” representing “definitely.” Any adverse event, such as muscular soreness, falls, or injury, will also be recorded by the research team.

##### 3.2.6.2 Semi-structured interviews

Semi-structured interviews will be conducted to assess participants’ perspectives and opinion regarding the multicomponent exercise intervention they received. The interviews will involve some broad open-ended questions about their experience and acceptability of the intervention. See Table 3 for the interview guide.

**Table 3:** Interview guide.

Interviewing questions for the perception of the multicomponent exercise intervention
1. How do you feel about the multicomponent exercise intervention? [probes: content, format, arrangement]
2. How would you describe your participation in this multicomponent exercise intervention?
3. Are there any barriers or hindering factors for your participation in this intervention? What are they?
4. Can you think of any strategies which can facilitate your participation in this intervention?
5. How does your health conditions change after engaging in this intervention? [probes: physical health, cognitive function, psychological health]
6. What are the possible reason for the changes in your health condition, if any? [probes: self-efficacy]
7. Does participating in this exercise training affect your daily life? Do your relatives and friends support your participation in this program?
8. Would you be willing to continue participating in this intervention program in the future? What are the reasons behind your decision?
9. Would you recommend this exercise program to your relatives and friends?
10. Is there anything else you would like to share with me about this exercise program?

#### 3.2.7 Safety protocol

Several measures will be implemented to ensure participants’ safety during the intervention. (1) Participants meeting exercise contraindications or assessed as high risk for exercise-related injuries will be excluded. (2) Instructions for the exercise intervention will include healthy advice to prevent injuries. (3) Prior to each exercise session, the exercise supervisor will conduct a brief medical safety check following ACSM recommendations. (4) If the participant reports concerning health changes, alterations in medications/dosage, or exhibits abnormal vital signs (e.g., blood pressure ≥ 180/100 mmHg), their participation in activities will be immediately paused, until they are seen and cleared to proceed by the study physician. (5) Participants will periodically report their perceived effort level on the Borg Rating of Perceived Exertion scale during each session to ensure proper exercise intensity. (6) There will be a warm-up routine before each session, and a cool-down routine after completion. (7) A multidisciplinary team (e.g., nurses, geriatricians, physiotherapists) has been involved in designing and implementing this trial to eliminate possible risks and prioritize participants safety.

#### 3.2.8 Ethics and dissemination

This study was approved by the ethics committee of Central South University (reference number E202249) and will be conducted according to the ethical standards of the Helsinki Declaration. After thoroughly discussing potential risks and benefits, written informed consent will be obtained from all participants before inclusion.

The study protocol has been registered and can be obtained through the Chinese Registry website (registered on ChiCTR.org with the identifier ChiCTR2200058850). The findings of the study will be published in scientific journals to target a wide range of groups and be reported at international conferences in the field of gerontology. Additionally, the results will be disseminated among study participants, healthcare professionals, healthcare providers and the public through courses, presentations, and online platforms.

#### 3.2.9 Data management and security

All potential participants will be assigned a unique ID number at the start of the assessment to maintain the participants’ confidentiality. Any handwritten information such as written consent forms or other related research materials will be stored securely in a locked cabinet when not in use. All electronic data will be securely stored on a server within the principal investigator’s institute. All data will be analyzed anonymously.

#### 3.2.10 Data analysis

Summary statistics, including means with standard deviations, frequencies, and percentages, will be generated for baseline characteristics. To compare the baseline characteristics between the two groups, Student’s *t*-test or the Wilcoxon rank-sum test will be utilized for continuous variables, while Chi-square or Fisher’s exact tests will be employed for categorical variables. Analysis of covariance (ANCOVA) and generalized linear models (GLM) will be performed to compare mean changes in continuous variables between the two groups. Adherence, dropout rates, program satisfaction, and adverse events will be tabulated and summarized using descriptive statistics. Mediation models will be used to test whether exercise self-efficacy mediates the relationship between the multicomponent exercise intervention and physical frailty, cognitive function, body composition, senior fitness, mental health, sleep quality, and health-related quality of life. Any missing data on outcomes will be approximated through multiple imputation. All analyses will be performed with the intention-to treat (ITT) principle. Two-sided tests will be performed for all analyses, and *p*-value less than 0.05 will be considered to be statistical significance. Additionally, sensitivity analyses will be adopted to assess the robustness of the findings based on the per-protocol analysis set.

Regarding qualitative data, audio-recorded interview will be transcribed word-for-word. Content analysis will be adopted to code the data on their perceptions of the intervention. Two authors will independently code the data, and any discrepancies will be resolved through discussion.

#### 3.2.11 Validity and reliability

A panel of six experts, encompassing fields such as geriatric nursing, sports medicine, exergaming, and public health, reviewed the study protocol. The protocol was finalized after making necessary modifications as suggested by the panel.

This study will adopt a stringent experimental design to enhance the internal validity of the study findings. The randomization of participants into different study groups will minimize the selection bias. Additionally, the characteristics of the study sample will be compared to determine any significant differences at baseline. The use of instruments with good validity and reliability will further enhance the credibility of the study findings.

Regarding qualitative data, interviews will be audio-taped to enhance trustworthiness. Two authors will independently code the data, comparing their coding to identify and resolve any discrepancies through discussion.

## 4 Discussion

As the global population ages, the number of older adults with cognitive frailty is rapidly increasing and expected to escalate in coming decades. This trend could result in an increased burden on families, caregivers, and healthcare systems (Kelaiditi et al., 2013). Consequently, the concept of healthy aging has been widely advocated (Keating, 2022). The World Health Organization (WHO) defines “healthy aging” as “the process of developing and maintaining the functional ability that enables wellbeing in older age”(Keating, 2022). Physical activity is one of the most crucial determinants identified for healthy aging (Abud et al., 2022). Substantial evidence supports the beneficial effects of exercise program in promoting health aging.

While exercise can yield numerous benefits for older population, not all forms of exercise offer equal health benefits. Exergaming, an aerobic exercise that combines cognitive stimulation and physical exercise, has shown specific potential in preventing cognitive decline (Zhao et al., 2020). Neurologic research demonstrates that older adults engaging in exergaming exhibit enhanced neuroplasticity, reducing the risk of developing dementia (Stojan and Voelcker-Rehage, 2019). Moreover, a recent systematic review indicated that exercise intervention positively impacts global cognition in individuals with mild cognitive impairment or dementia only when aerobic exercise is included in the program (Venegas-Sanabria et al., 2022). However, resistance exercise is the most effective method for enhancing muscle strength and physical functionality and should be prioritized within exercise program delivered to the older people living with physical frailty (Hurst and Sayer, 2022). Multicomponent exercise programs are highly recommended for older adults (Venegas-Sanabria et al., 2022). Considering cognitive frailty as a geriatric syndrome characterized by the simultaneous presence of cognitive impairment and physical frailty, a multicomponent exercise program that integrates exergaming and resistance exercise have the potential for simultaneously enhancing cognitive and physical functionality, maximizing overall health benefits among this vulnerable population.

To the best of our knowledge, this is the first mixed methods research to evaluate the effectiveness of a motivating multicomponent exercise, to explore the potential mechanisms of intervention effects among older adults with cognitive frailty, and to explore their perception of this program. By integrating exergaming into the program, we anticipate an increase in exercise self-efficacy and adherence to the physical exercise intervention, reinforcing the proven benefits of the exercise in this vulnerable population. The findings of this study could hold significance for public health and social policy. It is expected that the results of this research will guide clinical practice in community settings, so that clinicians and policymakers can provide more evidence-based practice for the health promotion for this vulnerable population.

## 5 Limitations

There are potential limitations that should be acknowledged. Firstly, although a large number of assessments give richer information about the study, the number of tests may pose the risk of missing data and dropout. Secondly, due to funding constraints, this study will only examine the effects over a 12-week period and will not include laboratory measurements (e.g., blood chemistry analysis). Future research that explores the long-term effects of the program and includes clinical measures and biomarkers testing in these samples are recommended. Thirdly, this study employs a single-blind design, with the data collector being blinded. However, blinding of the participants and interveners is not feasible. It is important to acknowledge that potential biases could arise from performance or expectation.

## 6 Conclusion

Due to the rapid aging of the population and the limited treatment options for disability and dementia, it is essential to promptly explore effective strategies to alleviate the considerable burden on the health and social care system. The proposed physical exercise intervention is based on a strong theoretical foundation and follows international recommendations for prescribing exercise in older adults. The findings of this study are expected to contribute meaningful knowledge about the beneficial effects of this novel exercise program, which will promote better functionality, independence, and improved quality of life for older adults with cognitive frailty.

### What problem will the study address?

In the field of exercise intervention for older adults with cognitive frailty, there exist major problems such as low adherence to the proposed program, reliance on a singular form of exercise, and limited exploration of the mechanisms underlying intervention effects. This study aims to address these challenges by conducting a mixed-methods research initiative among older Chinese community-dwelling people with cognitive frailty. The study will explore a novel multicomponent exercise training program designed to enhance both adherence and experience to physical exercise in this vulnerable group. Moreover, this intervention program, founded on the integrated Health Belief Model (HBM) and Self-Efficacy Model (SEM), seeks to elucidate the underlying mechanisms of the program. This study provides an effective evaluation of the exercise program from a broad perspective, including physical frailty, cognitive function, muscle-related outcomes, physical functional abilities, quality of life, senior fitness, sleep quality, and mental health.

## Data availability statement

The datasets presented in this article are not readily available because the waiver of consent and our IRB approval does not allow us to share the dataset used, without appropriate modifications. Requests to access the datasets should be directed to the corresponding author.

## Ethics statement

The studies involving humans were approved by the ethics committee of Central South University. The studies were conducted in accordance with the local legislation and institutional requirements. The participants provided their written informed consent to participate in this study.

## Author contributions

HN: Conceptualization, Methodology, Software, Writing – original draft, Writing – review & editing. FC: Conceptualization, Data curation, Investigation, Resources, Writing – original draft, Writing – review & editing, Supervision. JL: Conceptualization, Methodology, Supervision, Visualization, Writing – review & editing. YD: Conceptualization, Methodology, Resources, Supervision, Writing – review & editing. XC: Data curation, Investigation, Resources, Writing – review & editing. SW: Investigation, Visualization, Writing – review & editing, Methodology. AJ: Resources, Writing – review & editing. YG: Data curation, Formal analysis, Resources, Writing – review & editing. ZC: Methodology, Resources, Supervision, Writing – review & editing. HF: Conceptualization, Funding acquisition, Methodology, Project administration, Supervision, Writing – review & editing.
