# Corrigendum: Effectiveness of a multicomponent exercise intervention in community-dwelling older Chinese people with cognitive frailty: protocol for a mixed-methods research

**DOI:** 10.3389/fnagi.2024.1408556

**Published:** 2024-04-11

**Authors:** Hongting Ning, Fenghui Chen, Junxin Li, Yan Du, Xi Chen, Shuang Wu, Abigael Joseph, Yinyan Gao, Zeng Cao, Hui Feng

**Affiliations:** ^1^Xiangya School of Nursing, Central South University, Changsha, Hunan, China; ^2^School of Nursing, Johns Hopkins University, Baltimore, MD, United States; ^3^Nursing School, Xinjiang Medical University, Urumqi, China; ^4^School of Nursing, The University of Texas Health Science Center at San Antonio, San Antonio, TX, United States; ^5^Department of Epidemiology and Health Statistics, Xiangya School of Public Health, Central South University, Changsha, Hunan, China; ^6^Department of Physical Medicine and Rehabilitation, Xiangya Hospital, Central South University, Changsha, Hunan, China

**Keywords:** exercise intervention, exergaming, resistance exercise, community-dwelling, older adults, cognitive frailty, mixed methods

In the published article, there was an error regarding the author list. Hongting Ning and Fenghui Chen were not designated equal and first authorship. Hongting Ning and Fenghui Chen contributed equally to the work and share first authorship.

In the published article, there was an error regarding the affiliation for Fenghui Chen. As well as having affiliation 1, they should also have 3Nursing School, Xinjiang Medical University, Urumqi, China.

The authors apologize for these errors and state that they do not change the scientific conclusions of the article in any way. The original article has been updated.

